# Content-Based VLE Designs Improve Learning Efficiency in Constructivist Statistics Education

**DOI:** 10.1371/journal.pone.0025363

**Published:** 2011-10-05

**Authors:** Patrick Wessa, Antoon De Rycker, Ian Edward Holliday

**Affiliations:** 1 Leuven Institute for Research on Information Systems, University of Leuven, Leuven, Belgium; 2 School of Communication, Taylor's University, Subang Jaya, Malaysia; 3 Aston Brain Centre, School of Life and Health Sciences, Aston University, Birmingham, United Kingdom; Universidad Veracruzana, Mexico

## Abstract

**Background:**

We introduced a series of computer-supported workshops in our undergraduate statistics courses, in the hope that it would help students to gain a deeper understanding of statistical concepts. This raised questions about the appropriate design of the Virtual Learning Environment (VLE) in which such an approach had to be implemented. Therefore, we investigated two competing software design models for VLEs. In the first system, all learning features were a function of the classical VLE. The second system was designed from the perspective that learning features should be a function of the course's core content (statistical analyses), which required us to develop a specific–purpose *Statistical Learning Environment* (SLE) based on *Reproducible Computing* and newly developed Peer Review (PR) technology.

**Objectives:**

The main research question is whether the second VLE design improved learning efficiency as compared to the standard type of VLE design that is commonly used in education. As a secondary objective we provide empirical evidence about the usefulness of PR as a constructivist learning activity which supports non-rote learning. Finally, this paper illustrates that it is possible to introduce a constructivist learning approach in large student populations, based on adequately designed educational technology, without subsuming educational content to technological convenience.

**Methods:**

Both VLE systems were tested within a two-year quasi-experiment based on a *Reliable Nonequivalent Group Design*. This approach allowed us to draw valid conclusions about the treatment effect of the changed VLE design, even though the systems were implemented in successive years. The methodological aspects about the experiment's internal validity are explained extensively.

**Results:**

The effect of the design change is shown to have substantially increased the efficiency of constructivist, computer-assisted learning activities for all cohorts of the student population under investigation. The findings demonstrate that a content–based design outperforms the traditional VLE–based design.

## Introduction

In recent years, there has been a lot of interest in Computer Assisted Learning (CAL) in the academic community [1]. Some pedagogical studies however, take the system design of the Virtual Learning Environment (VLE) for granted – for example the study by Stricker, Weibel and Wissmath [2] investigated the impact of the VLE on learning outcomes without considering the possibility that software design may play a role of importance. This is surprising because the efficiency of CAL may be strongly influenced by the VLE's design [3] which is typically beyond the control of the educator.

In this paper we investigate whether a general purpose VLE design, providing learning resources and activities at the level of the management of the course of instruction (course–centered), is less efficient at promoting effective learning than one that is designed so that these features are adapted to the subject studied (content–centered). Measureable differences in learning outcomes between course–centered and content–centered VLE designs are what we call in this paper (VLE) design effects.

This study aims to demonstrate that the design effect of the VLE is indeed measurable and potentially substantial. In order to achieve this goal, a two–year comparative study was set up within the context of an undergraduate statistics course that was embedded in a pedagogically constructivist setting. Since there are no clear-cut definitions available in the literature [4], we define “constructivism” from a pragmatic point of view, without the intention to take part in the academic debate about educational theory: Constructivism is a theory that claims that deep learning takes place during a learner's active involvement in guided learning activities and with a certain degree of freedom and self-control. Knowledge is not the result of rote memorization but “constructed” from individual and social experiences which are triggered by guided learning activities that stimulate interaction, communication, experimentation, discovery, organizing, and conceptualization. In this sense the educator plays an active role as coach and facilitator, and the learner is expected to take up responsibility for the learning process. While the facilities that are made available in the VLE may play an important role in a general constructivist setting, they are of crucial relevance in our study as is explained in the next subsection.

### Historical Background

In the last six years, we have investigated ways to improve the quality of our statistics education. More precisely, we designed and created web–based technologies and built several educational applications to support students in their attempt to learn statistics. Our ultimate goal is to achieve a situation where students are able to learn and (truly) understand statistical concepts and associated methods at a deep level, as opposed to the rote memorization practices that we observed in the past.

In our experience there are effective ways to achieve non-rote learning. For instance, it is possible to achieve fairly good results by using techniques such as direct instructor–student interaction, in–class debate, worked examples, individualized instructor feedback about problem–based assignments, computer labs, etc. Unfortunately, these teaching approaches involve a lot of time and effort on the part of the instructors. Moreover, due to externally imposed temporal, physical, and monetary constraints, it is not feasible to employ such teaching approaches in undergraduate statistics education with large student populations. In order to compensate for some of these constraints, we soon started to search for innovative, technological solutions to overcome these barriers to improved statistics education.

Even though the use of educational technology (such as a VLE) does not guarantee educational success, there are a couple of promising, CAL approaches which seem to correspond to the student–centered learning vision of our universities (Aston University, University of Leuven). A student–centered, constructivist, approach to education places more responsibility on the shoulders of the student; on the other hand, it also implies that the instructor should play the role of “facilitator” and “coach”, rather than the person who simply reads the lectures and dictates the course requirements. One of the possible consequences of such an approach is that the instructors may be required to create a learning environment in which students get individual feedback about their performance, preferably on a regular (weekly) basis. This requirement, however, intensifies the tension between the student–centered learning approach and the constraints that are imposed by the institution.

One of the more promising learning tools that caught our attention is Peer Review (PR) because it can be supported by cost–effective software technology and because it is firmly rooted within the pedagogical paradigm of constructivism and is compatible with a student–centered learning approach [5]. The feedback that is generated by students may be beneficial for the receiver (reviewee), provided that there is a mechanism that ensures the quality of the feedback and under the condition that the PR process does not prevent students from experimenting [6]. More importantly, the reviewer may experience even greater learning benefits [7] if the quality of the submitted PR messages is graded by the instructors [8].

This explains why we decided to define PR, loosely aligned with the concepts described by Strijbos and Sluijsmans [9], as *the cyclical and iterative process of communicating relevant, well–argued and constructive feedback messages by students about the workshop papers of their peers*. This definition emphasizes that we view PR as a constructivist learning activity (which is graded by the instructors) rather than an exercise in which students grade each other. In addition, this definition is compatible with our goal to improve statistical understanding through constructivist learning, which is mediated by computer software.

For the above reasons, we introduced a series of problem–based workshops with a computer–supported PR mechanism in our undergraduate statistics courses. Based on our innovative software technology, it was possible to use use PR as a constructivist learning activity which promised to contribute towards non–rote learning [6]. The introduction of this PR–based approach, however, also raised important questions about the appropriate design of the Virtual Learning Environment (VLE) in which such an approach is implemented because the traditional VLEs (such as BlackBoard™, Moodle™, etc.) presume that any such learning activity can be simply plugged into the course–based structure of the system.

For this reason we decided to investigate two competing software design models for VLEs. Each model was implemented in identical course settings (i.e. goals, instruction, instructors, materials, lecture rooms, …). In the first system, all learning features (such as PR) were a function of the classical VLE. In contrast, in the second system, an alternative software design was used which incorporated learning features (such as forums, and PR–functionality) in the course's core content (the workshop documents which contained the statistical analyses). The difference between both systems has nothing to do with the functionality of the software but with the arrangement of the various components as is explained, in detail, in the Course Organization section.

### Virtual Learning Environment

The typical, modern VLE integrates a wide variety of general-purpose CAL techniques which are clustered around a course by design [10]. In this sense the VLE is supposed to be of a generic and course-centered nature. While there may exist many reasons why such a design is beneficial, there are no guarantees that such VLEs are well-suited to build effective and efficient learning environments in the field of statistics. One of the reasons for this is the fact that statistics courses may involve statistical computing which is not readily available in contemporary VLEs. As a consequence, educators may rely on external statistical software products which are often hard – if not impossible – to seamlessly integrate into the VLE. It is not surprising that some statisticians have found it necessary to develop user interfaces (such as R Commander [11]) or entirely new statistical software for the purpose of building a specific–purpose Statistical Learning Environment (SLE).

A nice example of such an SLE is called *Koralle* (an example-oriented software package for the purpose of correlation analysis). It has been used in pedagogical research such as [12] where it is explained why it is important to create statistical software that incorporates CAL features which are normally featured in the VLE. In their study it is argued that providing worked examples alone is not enough to achieve true understanding of statistical concepts. Students need to be explicitly challenged to engage in processing information and finding explanations. They also argue that true understanding cannot be achieved without feedback and collaborative learning. This example illustrates the tension between general-purpose VLE design and the specific-purpose SLE which envisions the integration of CAL features (such as communication, collaboration, feedback) with statistical computing.

In this study the standard VLE design is represented by Moodle [13] which is well-known in the academic community [14], and has been designed within the pedagogical paradigm of constructivism which is described in the literature [15], [16], and [17]. There are some important reasons why Moodle was the VLE of choice in our study:

- Moodle is a free and open-source product which allows researchers to make use of the underlying database for data mining purposes [14]
- Moodle provides many features that relate to social constructivism (such as Peer Review)
- Moodle allows the educator to specify external hyperlinks with embedded user–identifying tokens (this allows us to identify which students use the external statistical software)

Within the context of this study, the design effect that is investigated relates to the arrangement of the software components (Lego bricks) that support the socially constructivist learning activities within the VLE. In terms of the Lego metaphor, the design change comes down to creating, with exactly the same Lego bricks as before, an entirely new object with new (and hopefully better) properties as compared to the original object. Hence, the design change was obtained by removing the Moodle PR module and replacing it with newly developed (but otherwise equivalent) peer review software which was embedded in the Statistical Learning Environment as is outlined in the next subsection.

### Statistical Learning Environment

The key technological enhancement applied in the design of the SLE in both years was the incorporation of means by which statistical analyses could be reproduced, modified and re-distributed to peers. Indeed, the inability of scientists to reproduce published empirical research has received a great deal of attention within the academic community: [18], [19], [20], [21], [22], [23], and [24]. Several solutions have been proposed in [22], [24], and [25] but have not been adopted in educational research because of their inherent impracticalities. For this reason, we developed an innovative Compendium Platform (CP), which is hosted at http://www.freestatistics.org [26]. The CP allows us to create constructivist learning environments which are based on *Reproducible Computing* as described in [27] and [5] (and which is freely available at http://www.wessa.net and http://www.r-project.org; [28]). The CP has several advantages that relate to the monitoring of actual learning processes and educational quality control ([29]). Henceforth, the term SLE refers to the computational system that we created and which comprises the actual statistical software (*R Framework*), the CP (and associated repository of reproducible computations), and all interfaces that allow users and other software systems to interact with the components that are contained therein.

In other words, the SLE allows students and educators to create documents that contain statistical computations that can be reproduced by any reader through a simple web browser and an internet connection. The reader simply clicks the hyperlink of the computation and receives all meta information that is associated with the computation. This allows users to inspect every detail of the computation (including the underlying source code, data, parameters, etc.) and empowers them to recompute or re-use the computations – even if the parameters, datasets, or algorithms are changed ([5]). Creating, reproducing, and reusing computations contained in a reproducible document (*Compendium*) is easy and does not require any technical skills, nor understanding of the underlying R code. In addition, the use of *Compendiums* does not require users to download or install anything on the client machine (all computations are server–based).

All computational activities that are performed within the *R Framework* and CP are stored in a process measurements database. Therefore it is possible to investigate learning behavior (statistical computing, reproducing results, archiving results, searching the archive, etc.) of students based on objective measurements that are otherwise unavailable. For example, in the Results & Discussion section, it is shown that such measurements allow us to build statistical models that describe the relationship between discretized learning outcomes and objectively measured CAL activities (submitting feedback in peer review, or generating reproducible computations) based on *Reproducible Computing* technology which is described in [5].

## Materials and Methods

Because our study is based on experimental research with human subjects, we start this section with information about ethical considerations. Furthermore, we provide details about the practical organization of the statistics course under investigation. This is necessary to understand: *what* we did in the course, *how* the observed data are related to the learning process, and *why* the system design change is relevant for the students and their learning.

From a methodological point of view, we decided, for a variety of reasons, to use an experimental design with a unique combination of properties which is not typically found in software design studies ([30], [31]). The key characteristics of the experiment (i.e. the focus on learning efficiency, the quasi-experimental design, the equivalence of the control and the treatment group, the four cohorts, the two-years time span, the control of extraneous variables, absence of prior knowledge, and the multiple pretests that were obtained at weekly time intervals) have important reprecussions from a methodological point of view and need to be discussed in detail. Furthermore, it is necessary to explain how the learning outcomes are defined and statistically treated, without falling into the trap of subjectively assigned weights (of exam questions) by the instructor. Finally, we describe the statistical analysis methods and explain how the categorization was performed. Note that the figures in this section serve to show the pedagogical implications of VLE design and the impact of the changes we made.

### Ethics Statement

All students in this study had the opportunity to indicate whether they wanted to participate in the experimental, computer-assisted learning activities or not. This was achieved through a selection menu (so-called “radio-buttons”) from within the VLE (the choices were stored electronically and could not be forged because the students were required to logon to the VLE). During the first lectures, students received detailed information about the experimental status of the computing systems under study.

If a student did not participate in the experiment, we discarded all the data from that student – even if the student changed his/her mind after a few weeks. In addition, we provided the students with the opportunity to complete the course requirements based on a standard textbook in statistics and a traditional exam. The number of students in this situation was very low (no more than eight students per year).

In most situations, an official approval by an Institutional Review Board (or Ethical Committee) is not required for educational research, as is exemplified by the exemption of “*(i) research on regular and special education instructional strategies, or (ii) research on the effectiveness of or the comparison among instructional techniques, curricula, or classroom management methods*” which is specified by the Federal Policy for the Protection of Human Subjects of the National Science Foundation in the U.S.A. (http://www.nsf.gov/bfa/dias/policy/docs/45cfr690.pdf). Moreover, the applicable law on human experiments (*wet inzake experimenten op de menselijke persoon*, 7 May, 2004, http://ppw.kuleuven.be/onderzoek /ethischecommissie/wet) is explicitly limited to *experiments which develop our understanding of biology and medicine* – in other words, the legislation does not pertain to educational research as is presented in this paper. Notwithstanding the fact that our research is exempt from the traditional ethical review, we would like to point out that our research was funded by an academic agency which involves a series of screening and monitoring procedures, and which is only granted under the condition that there is institutional support and permission to study the pedagogical effects of the technological innovations that are implemented in our experiment.

In addition, there are several other facts which are connected to ethical conduct during the study:

- The data we collected through the experimental software did not contain any sensitive information.
- All records were only identified through unique, anonymous numbers.
- As is required by local legislation, the grading system and the constructivist setting of the course was accepted by the departmental council (“Departementale Raad”) which includes student representatives.
- Many of our courses have practical sessions in which students are graded based on a combination of effort and result (so-called “permanent evaluation”). The data that we collected in our study was very similar to the data that is commonly used in permanent evaluation.
- The collected observations did in no way cause students to be evaluated differently than without our research. For instance, we measured the number of blogged computations – this was only used for the purpose of research and never had an impact on student's grades.
- We spent a considerable amount of time explaining the grading system and data treatment to the students enrolled on the courses.

Finally, we did not employ a “fully randomized” experiment for our study because of ethical considerations. This is explained in more detail in the Reliable Nonequivalent Group Design subsection.

### Course Organization

**Figure 1:**
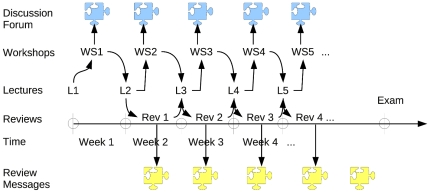
Schedule of learning activities – Year 0 and 1.

The empirical evidence we report here was based on an undergraduate statistics course for business students with a strong emphasis on constructivism. The course contained a wide variety of statistical techniques and methods such as: explorative data analysis, hypothesis testing, multiple linear regression, and univariate time series analysis. A total of 73 different types of statistical techniques were covered in the course, each investigated by students with a large variety of model parameters. For each technique, students had one or several web-based software modules available within the R Framework. In order to implement this course within a setting of constructivism for a large student population, it was necessary to impose a strict assignment–review mechanism. This is illustrated in Figure 1 which shows a series of weekly events (lectures, assignments, reviews) during a thirteen–week semester (the horizontal axis represents time). The semester ended with a final (open book) examination consisting of a series of objective multiple choice questions which referred to a 46-page document containing raw computational output (charts and tables about several data series). The examination was intended to test understanding of statistical concepts rather than rote memorization. More precisely, the exam was designed to test if students were able to:

- identify the computational output that was relevant to the question
- interpret the output in terms of the question
- critically investigate if the underlying assumptions of analyses were satisfied

These three learning objectives were explicitly included in the official curriculum description and explained to the students in the first lecture. In other words, student were informed about the fact that rote memorization (of statistical theory) would not increase their chances to succeed.

The main sections of the statistics course were built around a series of research-based workshops (labeled WS1, WS2, …) that require students to reflect and communicate about a variety of statistical problems, at various levels of difficulty. These problems have been carefully designed (and tested over a period of six years) and cannot be solved without additional information that is provided by the educator. Each workshop contained questions about common datasets and questions about individual data series provided to students – this dual structure of the workshops promoted both collaboration between students and individual work. The top (blue) puzzle pieces in Figure 1 represent threaded communication (between students) about each workshop.

Each week there was a (compulsory) lecture (labeled L1, L2, …) which was held in a large lecture hall that was equipped with computer screen projection and Internet facilities. With the exception of the first and last week, each lecture consisted of the following two parts:

- one or several illustrated solutions of the previous week's workshop assignment based on good and bad examples of archived computations that have been generated by students and the educator
- an introduction to next week's assignment including a reading list and an illustration

During each week, students were required to work on their workshop assignment and – at the same time – write peer reviews (labeled Rev1, Rev2, …) about (an average of) six assignments that were submitted by peers. Each review was based on a rubric of a minimum of three criteria and required students to submit a workshop score and an extended feedback message for each criterion. In Figure 1 these messages are represented by the bottom (yellow) puzzle pieces.

The grades that were generated by the peer review process did not count towards the final score of students. Instead, the educator graded the quality of the verbal feedback messages that were submitted to other students. The grading was performed based on a semi–random sampling technique which allowed the educator to grade the quality of a relatively small – but representative – number of submitted feedback messages from each student. The systematic part of the sampling process was based on various statistics that are automatically produced by the peer review software about the submitted assessment scores. Each review is accompanied by a score which can be easily compared to the scores that were given by other students. For instance, if five (out of a total of six) reviewers submit a grade which is “excellent” and only one student rates the work under review with a “poor” grade then this discrepancy can be immediately detected in the educator's overview screen which is created by the software. If such a case occurs, then the educator grades the quality of the feedback that accompanies the “poor” grade and two random feedback messages that correspond to “excellent” grades. This allows the educator to decide whether the “poor” grade was justified (e.g., the student discovered a serious problem) and whether the “good” grades have been given by students who did not properly analyze the document which was under review. More detailed information about how peer reviews can be reviewed by the educator is available in the study of [8].

As one might have noted, this feedback-oriented process is similar to the peer review procedure of an article that is submitted to a scientific journal. The process of peer review is an important aspect of scientific endeavor, and may help us in achieving learning goals with respect to attitudes (through peer review experiences) and skills (through construction of knowledge). The key idea behind this constructivist application is that students are empowered to interact with reproducible computations from peers and the educator. Students are required to play the role of active scientists who investigate problems, present solutions, and review the work of peers. Access to web-based *Reproducible Computing* technology is critical in allowing students to engage in such peer review activities.

#### Original System Design – Year 0

**Figure 2:**
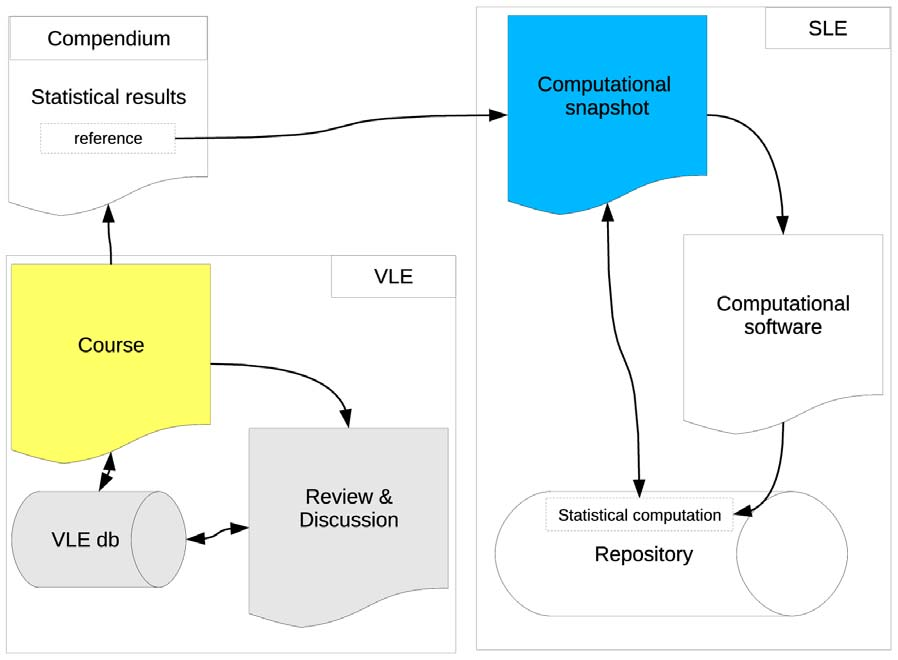
System Design – Year 0.

Figure 2 displays the VLE and SLE as they were used in Year 0 (2007 Fall Semester). It can be seen that this design contained two core objects: the course (yellow) and the computation (blue) which is represented by its snapshot. The course is the core object of the VLE which implies that all features that allow students to engage in collaboration or communication are bound to the course in which they reside. Several forums and instant messaging facilities were available to ask questions or to collaborate in various ways. In addition, the Peer Review & Assessment procedure was available from within the VLE – this includes all the necessary features that allow students to:

- obtain detailed information about the assignment
- electronically submit the completed assignment documents by the scheduled deadline
- obtain a list of peer submissions that are to be reviewed
- grade documents from peers (based on various rubrics) and send extended feedback messages about the peer's documents
- view and comment reviews that have been received

There are, however, several pedagogical problems with this type of design because students were unable to:

- engage in review activities when they viewed the meta information about a computation that was presented in a Compendium under review – instead they needed to login to the VLE and invoke the features of the Peer Assessment module
- read review messages that were submitted by other students about their own work unless they used the VLE and their own Compendium simultaneously
- compare review messages of computations that preceded the ones that were under review
- discuss or review statistical analyses across courses or semesters – as soon as the course was closed, all communications contained therein were lost forever

In addition, the collaborative communications about the workshops (blue puzzle pieces in Figure 1) and the feedback messages of the peer reviews (yellow puzzle pieces) were completely separated, which implies that working on assignments and learning through peer review were completely detached activities. Finally, and notwithstanding the fact that sequential workshops were related in various ways, there was no structural information about the dynamics of collaborative and review-based communications across workshops. For instance, if students were required to test a certain statistical assumption in an early workshop that was an essential condition to perform some type of analysis in a subsequent workshop, then there was no link between the communications of both. The only way that could have been used to solve this problem (within the Year 0 design) would be to repeat previous analyses in all related, subsequent workshops. Unfortunately, such an approach would have been highly inefficient and unfeasible because of many practical limitations.

#### Alternative System Design—Year 1

**Figure 3:**
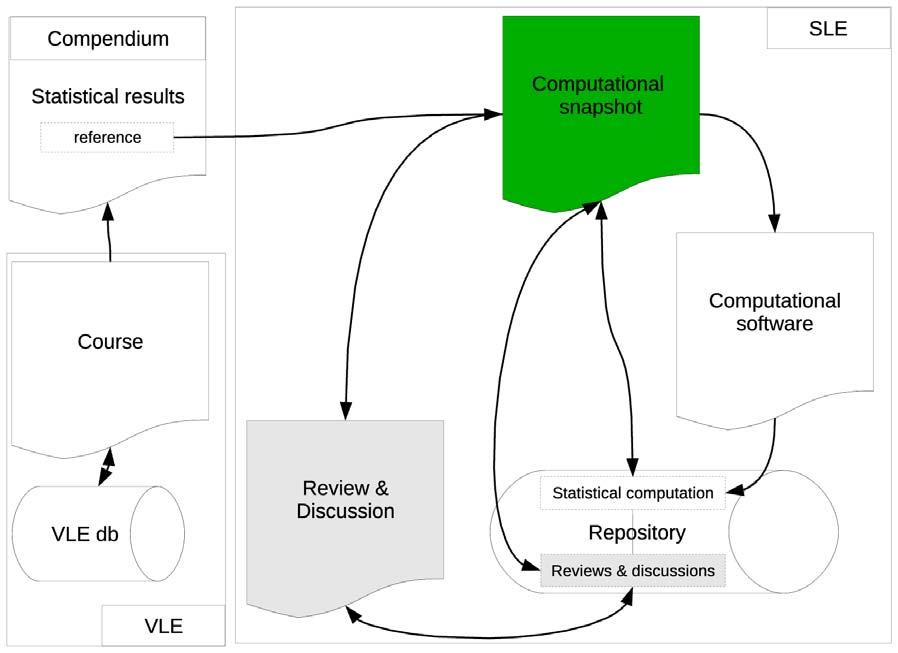
System Design – Year 1.

Figure 3 displays the alternative design that was implemented in Year 1 (fall semester of 2008). The most important design changes are as follows:

- there is only one core object: the computational snapshot (green object in Figure 3)
- all (threaded) collaborative communications about the workshops are available within the computational snapshot (which becomes a dynamic webpage)
- all review messages are associated with the computational snapshot

**Figure 4:**
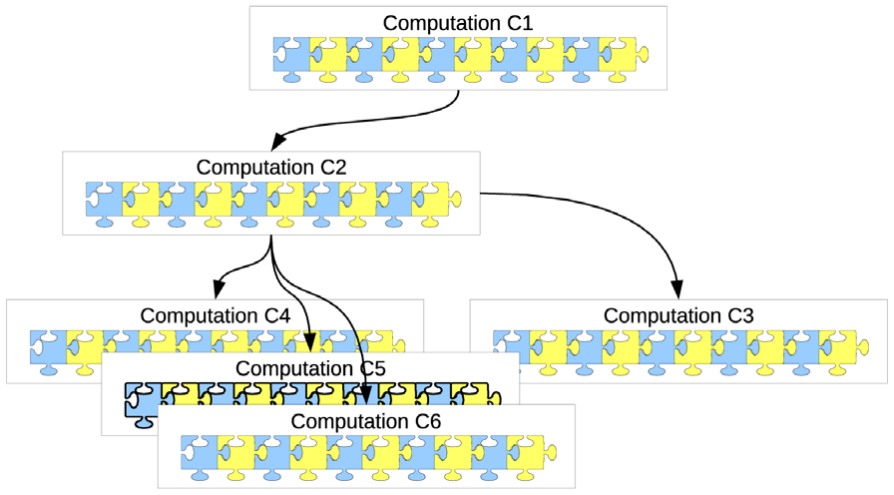
Hierarchical structure of computations – Year 1.

This design change had important consequences for the students because all collaborative and review–related communications were available from within the same source (the computation), which highlights how they are related – as is shown in Figure 4, the blue and yellow puzzle pieces within each computation are connected. This is not only true for a single computation – it also applies to discussion/review communications that relate to different computations, irrespective of the time frame, course, or workshop in which they originated. The reason for this is the fact that the CP automatically stores and maintains the parent–child relationships that exist between computations. For instance, if the educator creates a *Compendium* with a worked example that is based on an original computation C1 (see Figure 4) then a student may re–use this computation (with changed parameters or data) for the purpose of working on an assignment task (C2). At a later stage, the same (or any other) student may reproduce C2 (and create C3) in order to check the assumptions of a statistical analysis that is embedded in a subsequent workshop. Other students (across courses and years) may re–use C2 for similar purposes (computations C4–C6). Every time when a new *child computation* is generated (e.g., C6), its associated family tree is included in the meta data of that computation (which is also shown on the snapshot webpage). All parents (in this case C2 and C1) are automatically updated to include the new *child computation*.

The bottom line is that everyone who looks at C2 will have all the information that is available about computations C1–C6, including the hierarchical dependencies of computations and communications associated with them (the entire family tree is available). This design change should increase the efficiency by which users can gain an understanding of statistical concepts and the dynamics of how computations evolve (and improve) over time. Unlike in the traditional setting (Year 0) no information is ever lost after the semester because the communications are independent of the courses.

The fundamental principle that is applied in this system design is that the educational system is content–based instead of course–oriented. In statistics education, it is the statistical computation that is subjected to study – the course is entirely irrelevant. The traditional VLE is an educator–centered system that allows the educator to manage students, and resources that belong to the course. The new design is more student–centered because it focuses on the learning content which implies that all learning features (including communication, peer review, etc.) depend on the (content–based) core object.

### Methodology

The study conducted by Kampenes et al. [31] provides a nice overview of the use of quasi-experiments in software engineering. The authors found that 35% of all experiments (in their review of top software engineering journals) were quasi-experiments from which only 10% used the term quasi-experiment and only 8% mentioned the threat of selection bias. Even though the authors conclude that quasi-experiments are quite useful in software engineering research, they make a number of important recommendations from which we select the ones that are most relevant for our study, namely to:

- Examine whether the students in the control and treatment groups have the same characteristics:
  Do the students have the same curriculum history?
  Do the students have the same experience?
  What is the reason for students' availability at certain time points (years)?
- Use pretest measures and *nonequivalent dependent variables* to control for differences between experimental groups.

In addition [31], criticize the quality and lack of adequate reporting in a large proportion of the papers they reviewed. Hence, we make an attempt to provide a detailed report on how we took into account all of their recommendations.

#### Reliable Nonequivalent Group Design

The empirical data was collected through an experimental undergraduate statistics course which was provided during two consecutive years (labeled “Year 0” and “Year 1”) at a Business School of the K.U.Leuven Association in Belgium. In each year, the conditions that are under the control of the educator and the institution (such as: lecture rooms, educators, slides, lecture times, etc.) were kept equal except for the system design. This situation is commonly described as a quasi–experiment under the Nonequivalent Group Design (NEGD). It is well–known that this design has an internal validity threat which introduces a bias in the presence of measurement errors of the pretest – see [32].

Let 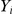 represent the exam score for 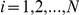 where 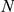 is the number of students. The degree of statistical knowledge before the course (the so–called pretest score) is represented by 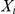 and is assumed to impact the exam score through the relationship: 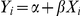. In addition, there is a binary treatment variable 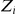 which is assigned a unit value if the subject 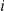 is in the treatment group (and a zero value if the subject does not receive treatment). The complete NEGD model becomes: 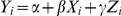 in which 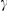 is the treatment effect that is subject to the classical hypothesis test 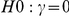 versus 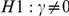. The NEGD selection bias (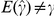) occurs when the (non–random) selection of subjects 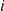 results in different average pretest scores 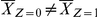 and in the presence of pretest measurement errors [32].

The statistical solution that is used to correct the selection bias is called Reliability–Corrected ANCOVA. Basically, the technique introduces an adjusted pretest score 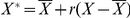 where 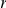 is the measure of reliability of the pretest. The estimate 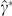 in 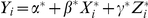 is unbiased if an appropriate estimate for 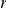 can be obtained.

In our study the NEGD bias was eliminated because students were known to have no prior knowledge (before the course onset) of the statistical concepts that were presented at the final exam (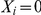 and 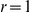). Note that those (few) students who had to re–take the course (and could have had prior knowledge) were excluded from the analysis. In other words the bias which would normally occur in such a NEGD is reduced to zero (and in addition both groups have exactly the same level of prior knowledge – i.e. zero). As is stated in [32]: “*Since measurement error on the pretest is a necessary condition for bias in the NEGD (if there is no pretest measurement error there is no bias even in the NEGD), if we correct for the measurement error we correct for the bias.*”

We made sure that there was no pretest measurement error by selecting an appropriate student population and a statistics course which involves knowledge and skills that are not available in the student population when entering the course. This is illustrated by the fact that students who did not actively participate in the learning activities but nevertheless made an attempt at the exam, failed and achieved extremely low scores. In order to emphasize the fact that this study's findings are not invalidated by the NEGD bias, we label the experiment as “Reliable” NEGD. Other types of internal validity threats (such as *Compensatory Rivalry* and the *History Threat* as described in [33] are also not likely to be present.

Notwithstanding the above arguments and in accordance with the recommendations of [31], we carefully investigated the characteristics of the treatment and control groups to identify any differences. All students (from both years) were required to submit three surveys (with a total of 101 questions) that attempted to measure their attitudes towards thinking and learning, learning experiences, and perceptions of software usability of the system. There were no significant differences between groups when the survey scores were compared item–by–item or when the analysis was based on constructs that are used in literature (connected learning, separate learning, peer support, interest in statistics, evaluation about the educator, ability to understand messages from peers or the educator, etc.). Some of the more important constructs that were used to assess equivalence between both groups are provided by measures within the Constructivist On-Line Learning Environment Survey (COLLES) [34]:

- Practical Relevance (of Statistics)
- Critical/Reflective Thinking
- Cognitive Demand (by Instructors)

To evaluate the similarity of the groups we computed independent T-Tests about the mean and Asymptotic Wilcoxon Mann-Whitney Rank Sum Tests for each construct between the two groups. The p-values of the T-Tests are shown in Table 1 and can be reproduced and verified with an R Framework application that was created (http://www.wessa.net/rwasp_vle_software_design_tests.wasp). The statistical results do not justify a rejection of the null hypothesis that there is a no difference between both groups. It should also be noted that the surveys are likely to be representative of the student population because the response ratio was very high (approx. 84%).

**Table 1:** Nonequivalent Dependent Variables Tests (http://www.wessa.net/rwasp_vle_software_design_tests.wasp).

Variable	Welch Two Sample	Asymptotic Wilcoxon
	T-Test (p-val)	Mann-Whitney Rank Sum Test (p-val)
Total Workshop Score	−0.2128 (0.8316)	−0.3289 (0.7423)
COLLES “Relevance”	−0.3483 (0.7278)	0.1173 (0.9066)
COLLES “Critical Thinking”	1.2576 (0.2092)	1.1916 (0.2334)
COLLES “Cognitive Demand”	0.9616 (0.3368)	0.6577 (0.5107)

Many of the constructs in these surveys serve as so-called *nonequivalent dependent variables* which strengthen our confidence that there are no structural differences between the control and treatment groups. Even though we *know* that both groups have no prior knowledge, we investigated the performance of students at each workshop that was submitted (peer assessment grade). As explained before, these weekly workshop scores did not count towards the final grade of students. However, they can be used as valuable *nonequivalent dependent variables* which allow us to conclude that there were no *time-varying confounding effects* in the control or treatment group. In other words, students in both years had on average the same workshop scores during the semester which strengthens our belief that their prior knowledge and intelligence is equal. The p-value of the corresponding T-Test for a difference in grade outcomes between the groups is 0.8316 (0.7423 for the Wilcoxon Test), which implies that the null hypothesis of “no difference in prior knowledge” cannot be rejected at any reasonable type I error level (see Table 1). This result can also be verified through the web-based software that we made available (http://www.wessa.net/rwasp_vle_software_design_tests.wasp).

In addition, there were no statistically significant differences as measured by: age, prior education, race, and scholarships. Students who had a special educational status (e.g. exchange students and athletes, who were not required to participate in weekly assignments) were excluded from the dataset. As it would be unethical to perform a truly randomized experiment (in which one group would potentially have an unfair, technological (dis-)advantage within the same course) our Reliable NEGD is the next best solution (within an academic year, the rules are the same for everyone). As a matter of fact, it could be argued that a truly randomized experiment would be worse than the Reliable NEGD because there is no practical way to physically separate the treatment and control groups of the same academic year. If these groups cannot be physically separated (during the entire period of the course) there is bound to be a psychological effect when students from both groups start making comparisons (this is called *Resentful Demoralization* in [35], [36]). Moreover, it would also be impossible to rule out contamination of students from the control group who start using the technology from the treatment group (this is called *Diffusion or Imitation of Treatment* in [33]).

Assessing the quality of learning systems relies not only on obtaining exam scores but also relates to the input of effort by the student. This is explicitly taken into account in our study and has important consequences. Let 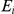 represent the objectively measured effort that is needed by student 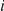 to learn through the use of the SLE that is made available (e.g., the number of statistical computations that is generated by the student). Considering the fact that 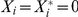 we can re–write the model to test the experimental design effect as follows: 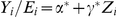 where 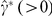 is the estimate of increased efficiency under investigation. In other words, instead of looking at the effect of system design on the level of exam scores we are primarily interested to find out if changing the design can improve the efficiency of CAL. It is our assertion that in both years, the active and bright students are equally likely to accomplish the learning task and pass the exam (no matter which technology is used) – after all, motivated and capable students will probably do whatever it takes to pass the exam. Therefore, we want to show that the design change allows students to achieve the same goals with less effort – the SLE is a tool which allows students to make progress more quickly or easily than would otherwise be possible. Considering the fact that learning efficiency is determined by educational technology and student's learning abilities, we may consider the following question to assess the internal validity of our experimental study: “Are students in Year 1 likely to be intrinsically more efficient learners than students in Year 0?” The data from the workshop scores and *nonequivalent dependent variables* indicate that the answer is negative.

For all of these reasons, we suggest that any changes in learning efficiency can be attributed to the change in VLE system design from the Year 0 to the Year 1 course. In addition, the study takes into account interaction effects which are associated with two different cohorts that are known to be relevant from previous research (e.g. [37]): bachelor students and students from the preparatory programme which allows graduates from a professional bachelor programme to switch to an academic master. In general, bachelor students have better prior understanding of mathematical concepts than prep–students. However, prep–students tend to have a higher degree of maturity and self–motivation than bachelor students. Finally, we also took into account gender differences for both cohorts which implies that a total of four subpopulations (as shown in Table 2) were used in each year [37].

**Table 2:** Number of students in the Reliable NEGD.

	Year 0		Year 1	
	Female	Male	Female	Male
Bachelor	58	53	41	42
Prep.	53	76	45	74
Total	240		202	

In summary, the experimental design that was used in this study has a unique combination of properties which is not found in the typologies used in review studies such as [30] and [31]. Its key characteristics are that: it focuses on learning efficiency (not only exam scores); it is a quasi-experimental design; there were two nonequivalent groups (one control and one treatment group); four cohorts participated over two years; many extraneous variables were controlled by the design (course content, lecture rooms, instructors, …); there was one posttest (the final exam); absence of prior knowledge was established on sound statistical evidence; data was collected from multiple pretests at weekly time intervals and that many *nonequivalent dependent variables* were defined.

#### Objective Exam Score Transformations

In order to be able to compare the dependencies of exam scores on exogenous variables, which are based on objective measurements of (constructivist) learning activities, it is necessary to apply optimal exam score transformations for both years. The methodology that allows us to do this is based on a mathematical model which is described in [29] that has been shown to yield statistical models that improve the predictability of learning outcomes substantially.

The methodology of objective exam score transformations involves three successive stages. First, a classical regression is used to predict the original exam scores as a linear function of 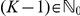 exogenous variables of interest. Let 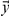 represent an 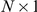 vector for all 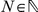 students (with 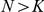), containing the weighted sum of 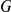 item scores (scores on individual exam questions): 
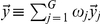
 with initial unit weights 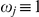. In addition, define an 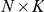 matrix 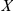 that represents all exogenous variables (including a one–valued column which represents the constant), and a 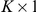 parameter vector 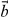 that represents the weights of the linear combination of all columns in 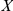 that is used to describe 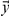. The complete model is denoted 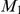 and is defined by 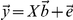 where 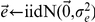 represents the prediction error.

In the second model 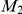, the prediction of the first model is specified by a linear combination of the individual items (questions) that made up the total exam score. Let 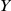 represent the 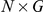 matrix that contains all 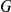 item scores, then it is possible to define the model 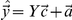 where 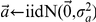. Note that there is no constant term in this model.

The third model (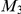) simply combines 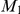 and 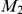 by relating 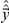 to 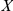 in the regression model 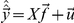. The estimator for 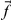 can be shown to be 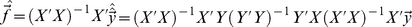.

In other words, the methodology of objective exam score transformations changes the weights that are attributed to individual exam questions in such a way that the predictability of the (transformed) exam scores (based on exogenous variables) is maximized, which implies that we are able to identify the parameters that are really important. Some of the main reasons why this is absolutely necessary are the following:

- the weights that are applied by the educator to each exam question (e.g. equal values) are arbitrary, whereas the above methodology yields objective weights
- some questions may have been poorly formulated by the educator, but after transformation, such exam questions will have an extremely low weight because they cannot be predicted by objective exogenous variables
- the educator changes the wording and structure of questions based on experience from previous years. This inevitably introduces biases which are avoided by objective exam score transformations

After the objective exam score transformation has been applied, it is possible to proceed to the next step which involves the creation of predictive models (regression trees) that allow us to discover the rules that seek to determine whether students will pass or not. In this study, the degree of predictability is maximized (through the transformation methodology) but is otherwise irrelevant to answering the main research question: “Does the changed system design introduced from Y0 to Y1 improve learning efficiency (through peer review) in the undergraduate statistics course?” In other words, we are mainly interested in the (efficiency–related) parameters of the decision rules, not the original (untransformed) exam scores (which cannot be legitimately compared), nor the overall degree of predictability.

#### Regression Trees

Regression Trees (RT) have been used in the field of software engineering for the purpose of detecting fault-prone modules or assessing software quality as is illustrated in [38], [39]. In particular, the use of association rule mining for the ongoing improvement of VLEs has been illustrated in [40]. The authors of the study argue that the proposed data mining tool can be used by non–expert instructors which allows them to make informed decisions about how the VLE can be improved. In addition, we argue that the rule-based RTs – belonging to the collection data mining methods – have several advantages when compared to classical statistical hypothesis tests within the context of the evaluation of educational technology and educational quality control:

- the rules of RTs are easily understood and support operationally feasible decisions
- there is no assumption about the functional (e.g., linear) form of the relationship
- RTs allow for non-monotonic relationships to be detected (this is not the case in multiple regression)
- the underlying assumptions are mild because tree methods are nonparametric
- RTs can be used even if there is little a priori knowledge about theories that relate the dependent and independent variables

RTs can be used as a tool for *Exploratory Data Analysis* and they have the ability to select relevant variables that are helpful in predicting the outcome of the dependent variable. This is particularly important in educational software engineering research because the developer/designer may not know how the learning outcomes may be affected by the use of the system. The main disadvantage of RTs is that the method easily leads to over-fitting. Cross Validation techniques have been advocated to detect such problems and have been implemented in most statistical analysis software featuring *Regression Tree Analysis* as described in [41].

The RTs employed in our study are capable of selecting the most relevant 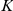 effort levels 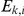 for 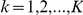 that are helpful in the prediction of the optimally weighted exam score. The statistical model is re-formulated in terms of rules like the following: 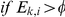 then 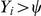 where 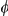 is an effort threshold level and 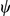 is the minimum exam score that is required to pass the exam. In other words, any student 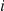 who generated more than 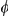 units of the 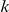
th learning activity (e.g., computations) is predicted to pass the exam.

If we want to determine if the design change had a beneficial effect, there are (possibly) several RT rules that can be examined and used to make a decision. Hence, the design effect is not reduced to one simple hypothesis test because that would assume that the quality of the system can be summarized in just one figure (which constitutes a highly unreasonable assumption – see [42]). Instead, we look at the threshold values (such as 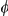) and hope that the new design yields lower treshold values than the old one. In addition, we need to determine if the RT rules make sense in terms of what we might tolerate (or wish for). For instance, it would be intolerable if girls would have no opportunity to pass the exam (regardless of their effort levels). In addition [42], also criticize classical approaches to treat selection bias on the grounds that they might be sensitive to violation of the underlying model assumptions. Hence, this is another reason why RTs are used in our analysis.

For the purpose of computing easily understandable, rule–based RTs, the endogenous variable must be discretized. Therefore, three categories are defined which are called “guess”, “fail”, and “pass” respectively. The “guess” category represents the lowest exam scores which can be attributed to chance or guessing. Exam scores in the “fail” category are lower than what is needed to pass the exam but higher than what can be reasonably explained by chance. The “pass” category contains scores that are sufficiently high to be considered satisfactory even if the numerical value is below 50% of the maximum attainable score. The reason for this is the fact that the exam questions had varying degrees of difficulty and were designed to be much more difficult than what could be reasonably expected from undergraduate students in business studies. Introducing a high degree of difficulty in the exam questions is necessary in order to ensure that:

- rote learners are not likely to pass the exam
- we are able to identify the maximum level of understanding
- students are unable to quickly find answers in printed resources that are allowed during the exam

The exam in the second year was slightly more difficult than in the first year (the transformed exam scores in Year 1 were slightly lower than in Year 0). Therefore it is not possible to simply use identical threshold values for the categories in the transformed exam scores from both years – an objective benchmark is need to generate fair and comparable categories.

The threshold values that define the categories are not arbitrarily chosen but depend on exam score statistics of the previous four years (with exams of similar difficulty). On average the proportion of lowest scores (which fall in the “guess” category) was little less than 10%. The proportion of “guess and fail” scores was approximately one third of all exam scores. These proportions were quite stable over the time frame of those four years. Therefore it is fair to assume that they represent appropriate, unconditional probabilities to pass or fail the exam. As a consequence the threshold values that define the three categories (for each year) are computed as the 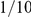 and 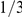 quantiles of the (optimally weighted) exam scores in Year 0 and 1.

Beyond our assertion that the threshold values are adequate, there is another justification for the same sample quantiles (rather than identical exam scores) to determine the categories. The rationale is simply that we want to predict if students fall in the “high”, “low”, or “extremely low” proportion of all students in the same year (who took the same exam). The parameters in the rule–based RTs quantify the learning efforts (number of peer review messages and number of computations) required to achieve an exam score that falls within the top 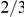 of all scores.

Rule–based RTs were computed with the statistical engine called *Weka* which is described in [43]. The functions of *Weka* are all available from within the R Framework through the *RWeka* interface developed by [44].

## Results and Discussion

The statistical computations in this section can be reproduced with the web-based software that we have made available. There is no need to download or install any software because all computations are performed remotely. The software, data, and analytical results can be found at the following URL: http://www.wessa.net/rwasp_vle_software_design.wasp.

### Results

As explained in the Materials and Methods section, we have provided a list of arguments and analysis to support statistical equivalence between both experimental groups. Some of the key variables that can be used to assess equivalence are highlighted in Table 1 – the results show that there are no differences between the students in year 0 and 1 if a type I error level of 20% (or lower) is used. Even if a higher type I error level would be chosen, the difference would imply a bias towards year 0, the year in which the traditional system design was used.

Table 3 shows the exogenous variables that were chosen to create rule–based regression trees. This choice was based on previous research (such as [29], [45], [46]) which allowed us to focus on the most important variables. The first three variables are positive, numeric integers. The last two variables are binaries that indicate to which cohort the student belongs. Note that the same exogenous variables were used in the objective exam score transformations based on the three–stage regression approach and with all possible interaction effects included.

**Table 3:** Nomenclature in rule–based regression trees.

Variable	Description
nnzfg	# of non–zero meaningful feedback
	messages given (by students)
nnzfr	# of non–zero meaningful feedback
	messages that were received
Bcount	# of reproducible computations
Gender	gender ( = 0 for females, = 1 for males)
Pop	binary cohort variable
	( = 0 for bachelor, = 1 for prep)

**Figure 5:**
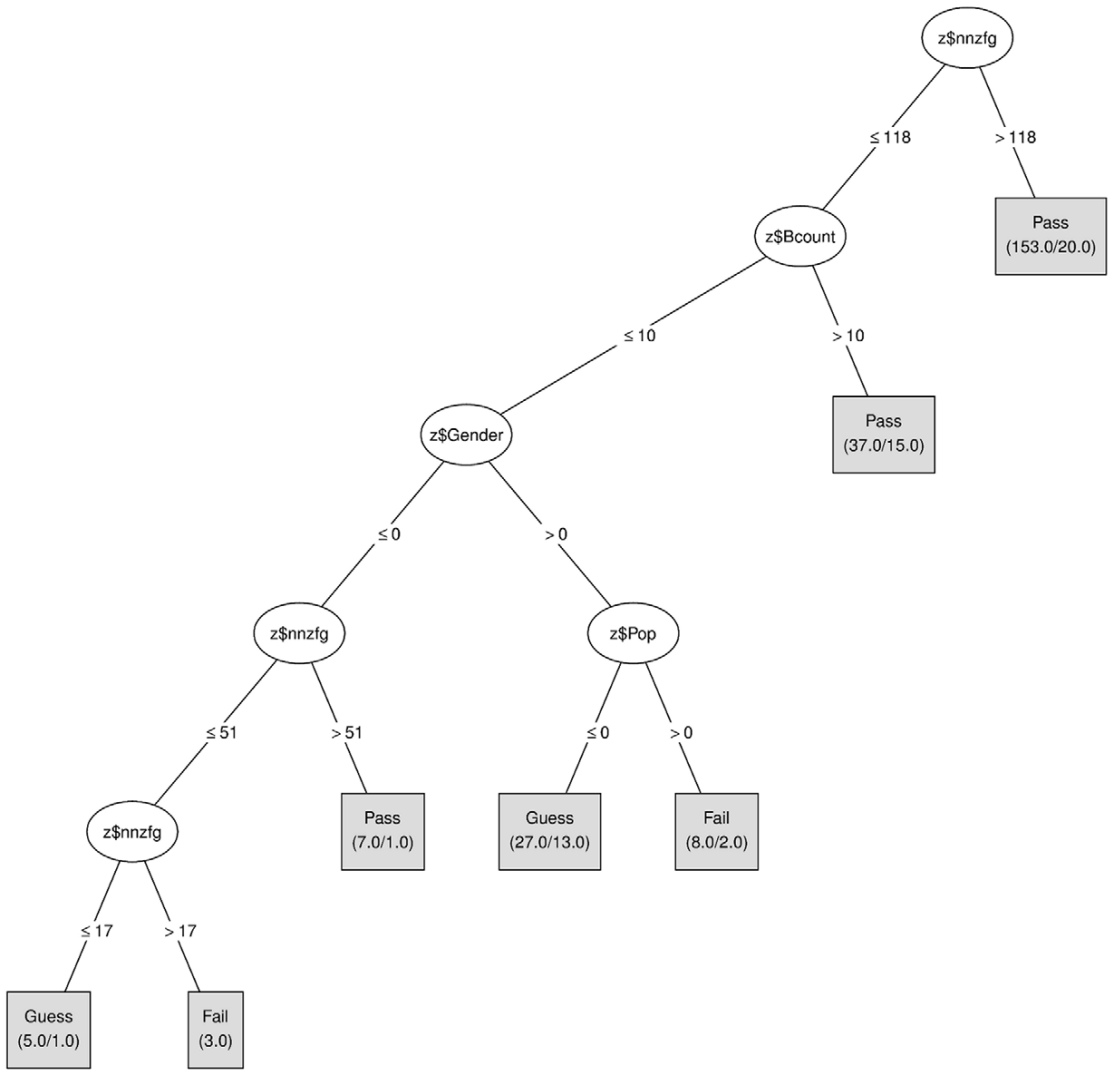
Regression Tree – Year 0. (http://www.wessa.net/rwasp_vle_software_design.wasp).

The first rule–based regression tree (see Figure 5) displays the situation for Year 0 in which the traditional VLE design is used. The most important rule that determines whether students “pass” (fall into the top 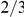 proportion of all students in Year 0) is the number of submitted feedback messages (which are related to peer review). It can be seen that students “pass” if 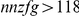 which means that they need to submit more than 118 meaningful feedback messages in order to pass the exam. The other students (with 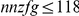) fall into two categories, depending on the number of reproducible computations they generated. Students with 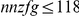 and 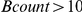 are predicted to pass the exam – in other words, students who did not write enough feedback messages could compensate this by reproducing more than 10 archived computations. However, the accuracy of this particular prediction is not very high: the model assigns 37 cases into the “pass” category from which 15 cases did actually fail (these numbers can be seen in the grey boxes).

There are two specific rules in the regression tree that demand our attention. The first one is the rule that states that male students who did not make a sufficient amount of effort in terms of feedback and reproducing computations (formally: 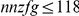 and 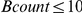 and 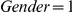) either fall into the “guess” or “fail” category (depending on the value of 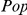). The second rule that causes concern is the one that states that female students may pass the exam, even if they have only between 52 and 118 submitted feedback messages (formally: 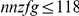 and 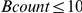 and 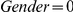 and 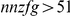).

The bottom line is that both rules imply that the system in Year 0 favors female students and discriminates against males. This may be surprising because there is some evidence to suggest that male students have more positive attitudes towards computing than females [47]. In this situation, however, it is shown that female students are better able to cope with the detached structure between collaborative and review–based communication on the one hand, and reproducible computing on the other hand. This phenomenon may have psychological causes that are related to the fact that there are gender differences in how students use communication in learning. Within the context of this study, such an explanation remains speculative and unanswered. However, and more importantly, it is clear that the segregated design of the VLE and SLE adopted in Year 0 (Figure 2) is not optimal – at least for an important part of the student population (roughly 20% of males).

**Figure 6:**
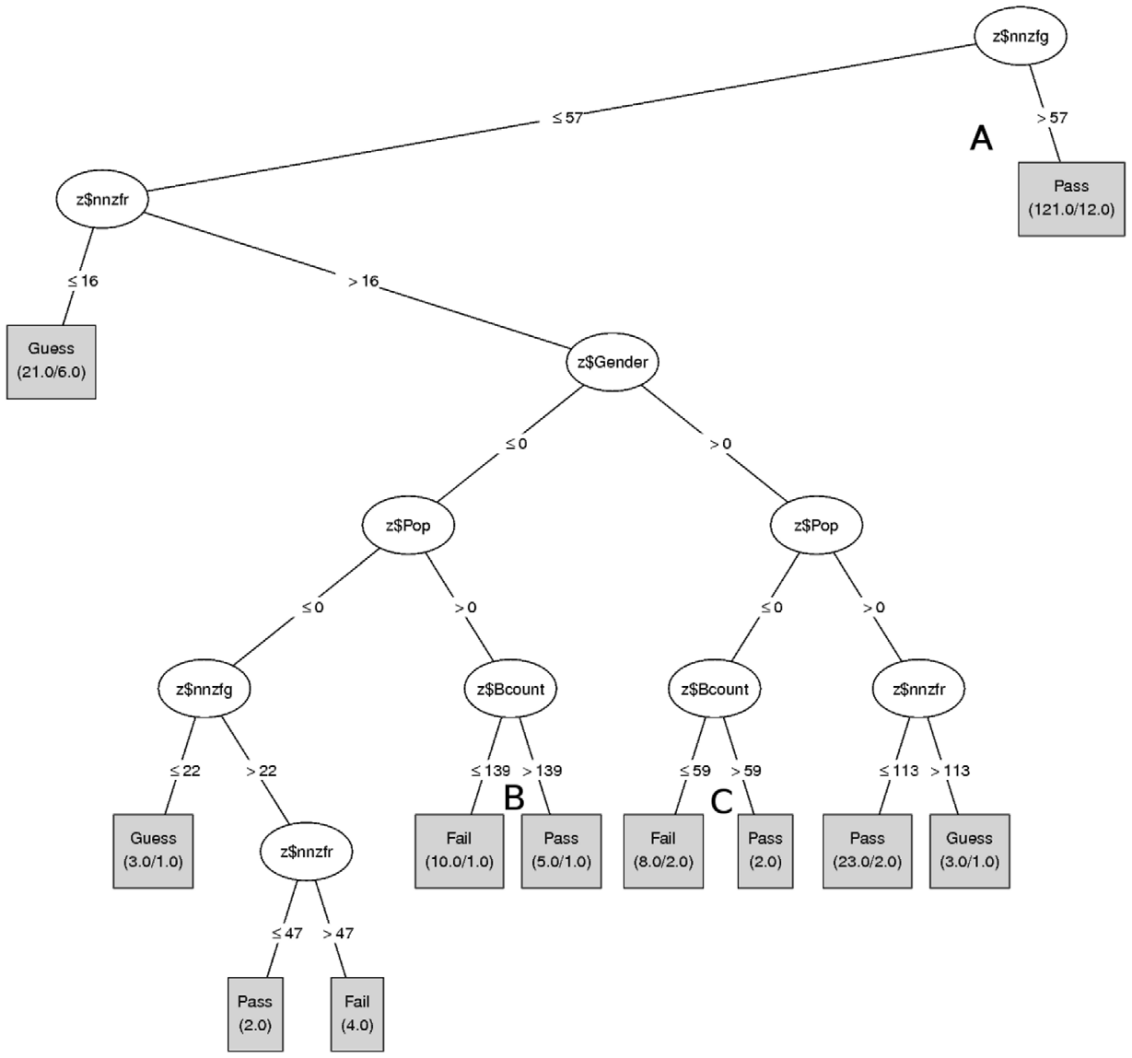
Regression Tree – Year 1. (http://www.wessa.net/rwasp_vle_software_design.wasp).

Figure 6 shows the rule–based regression tree for Year 1 (in which the new design was implemented). It can be easily observed that the structure is fundamentally different from the previous situation. By far, the most important property of this regression tree is the root rule which states that students pass if they submit more than 57 meaningful feedback messages (Label A in Figure 6). This is less than half the amount that was necessary with the previous system design and demonstrates a spectacular increase in review–based learning efficiency. More importantly, the gender discrimination effect has completely disappeared which implies that males are now equally well able to make good use of the learning environment (see values in corresponding boxes below the gender node).

Students who did not submit a sufficient number of feedback messages and only received 16 messages (or less) fall into the “guess” category. This makes a lot of sense because students who do not submit workshop papers, don't get reviews. Hence, these students simply did not participate in the assignment–review scheme that was outlined in Figure 1.

As explained before the overall predictability (of both rule–based RTs) is not critical in determining whether the design effect had any impact on learning efficiency. Nevertheless, an overview of within and out–of–sample prediction performance is provided in Table 4 because it is important to show that the models do not suffer from severe *over–fitting* which might invalidate all conclusions made on the basis of the RT's parameters.

**Table 4:** Within sample and Cross Validation prediction of RTs (http://www.wessa.net/rwasp_vle_software_design.wasp).

	Year 0		Year 1	
	Within	CV	Within	CV
Corr. Classif.	78.3%	72.5%	87.1%	74.8%
Incorr. Classif.	21.7%	27.5%	12.9%	25.2%
Leaves	7		11	
Tree size	13		21	
Cases	240		202	

The results in Table 4 illustrate that the out–of–sample prediction quality is adequate. In case of over–fitting, one would observe high percentages of correctly classified instances within sample and a (very) low percentage out–of–sample. The out–of–sample prediction quality is computed by applying a so–called Cross Validation technique which randomly divides the data set into a large training subset and a (smaller) testing subset. The parameters are estimated, based on the training sample, and the prediction is computed for the testing subset. This procedure is repeated 10 times (10–fold Cross Validation) to obtain an average measure of out–of–sample prediction quality.

### Discussion

An interesting observation can be made about the lower part of the regression tree that is shown in Figure 6 (labels B and C). There is a striking resemblance between female prep–students (B) and male bachelor students (C) because they both pass the exam when a sufficient number of computations have been reproduced. In addition, the female bachelor students and male prep–students are also similar with respect to the number of received feedback messages: if this number is too high, then the student does not pass because it indicates that they are making too many mistakes or are not making good use of inbound messages.

One might wonder why there is such a big difference between the threshold values that are associated with the Bcount variable. While this question remains unanswered in this study, there is a plausible explanation for this result. Based on focus group discussions, we know that most female prep–students seem to enjoy statistical computing (in comparison to other groups) whereas male bachelor students perceive statistical computing as a necessary but useful learning activity. In other words, female prep-students may have “more fun while being less efficient” in the way they use the results from statistical computing. This remarkable difference in threshold value does, however, in no way constitute concern from the software engineering point of view because:

- both students groups are able to pass the exam (there is no discrimination)
- bachelor students and prep–students are not in the same class - they mostly live separate lives on campus and do not compare learning conditions accross groups (which is confirmed by focus groups discussions)

More importantly, and based on Reliable NEGD data, the RT results presented above showed that the change in system design had a beneficial effect in terms of increasing the learning efficiency of submitting peer review messages. More importantly, the design change has resulted in the elimination of a previously unidentified gender difference which was present in the original design, where communication and computation were separated. Using the methodology outlined here, any software–related or content–based aspect of a VLE can be tested as long as it is controllable by the educator or designer of the learning system. However, one should always take care that exam scores are properly treated in the modelling process in order to avoid the pitfalls that are associated with exam questions, as we have done using the objective transformation method.

Our secondary objective in this research was to demonstrate the usefulness of PR as a constructivist learning activity which supports non-rote learning. This was demonstrated by the fact that the variable 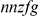, the index of PR activity, is the best predictor variable in both RTs. Students who submit more feedback messages increase their chances to perform well at the exam. Even though it is not possible to induce *causality* from this type of analysis (only a “predictive” relationship has been established at this point), this result indicates that our efforts to build learning environments based on PR technology were not wasted and warrant more in–depth investigation in the future. At the time of writing, we are conducting research that should allow us to demonstrate *causality* and measure the *effect-size* of the PR.

As a third objective, we established that it is possible to introduce a constructivist learning approach in statistics education, even if the student population is large and even if physical, temporal, and financial restrictions are imposed. The good news about this is that the technology is available to anyone wishing to improve educational quality. Our *Reproducible Computing* technology is already freely available – readers who are interested in using our PR technology are encouraged to contact us.

The findings from our experimental study have little generalizability beyond our undergraduate statistics course for business students. Also, the focus was on peer review, which leaves open the question whether other constructivist learning activities (e.g. problem–based learning) might have resulted in other conclusions.

Still, based on the evidence presented here it is interesting to formulate a general conjecture about a fundamental principle of good VLE design. The proposed conjecture states that good VLE design requires the developer to define a *content–based core object* instead of using the traditional, *course–centered core object*. In other words, it is better to integrate learning features (forums, messaging, peer review, etc.) into the software that delivers the subject under study (e.g. research documents and/or statistical software) than to re-engineer components of general–purpose VLEs in order that students can communicate effectively within a system that is primarily designed for course and student management.

Put differently, it seems reasonable to suppose that similar benefits of adopting this approach can be expected in disciplines other than statistics. In addition, and even though this study originated from a constructivist perspective, our findings may have implications that go beyond constructivist statistics education. Within the context of our conjecture, it is our assertion that there are three key properties that determine whether or not our findings are applicable to other courses and pedagogical approaches:

1. the learning process relies intensively on computer software (other than the traditional VLE)
2. the learning activities involve social interaction, collaboration, and/or communications
3. the learners are required to submit their assignments

If our conjecture turns out to be true, it would have important repercussions for the design of VLEs in general and specific–purpose software (statistical software, wikis, CAD/CAM applications, programming environments, etc.) in particular.
